# Validity Evidence for the Research Category, “Cognitively Unimpaired – Declining,” as a Risk Marker for Mild Cognitive Impairment and Alzheimer’s Disease

**DOI:** 10.3389/fnagi.2021.688478

**Published:** 2021-07-26

**Authors:** Rebecca Langhough Koscik, Bruce P. Hermann, Samantha Allison, Lindsay R. Clark, Erin M. Jonaitis, Kimberly D. Mueller, Tobey J. Betthauser, Bradley T. Christian, Lianlian Du, Ozioma Okonkwo, Alex Birdsill, Nathaniel Chin, Carey Gleason, Sterling C. Johnson

**Affiliations:** ^1^Department of Medicine, University of Wisconsin-Madison School of Medicine and Public Health, Madison, WI, United States; ^2^Wisconsin Alzheimer’s Institute, University of Wisconsin-Madison School of Medicine and Public Health, Madison, WI, United States; ^3^Wisconsin Alzheimer’s Disease Research Center, Madison, WI, United States; ^4^Department of Neurology, University of Wisconsin-Madison School of Medicine and Public Health, Madison, WI, United States; ^5^Madison VA GRECC, William S. Middleton Memorial Hospital, Madison, WI, United States; ^6^Department of Communication Sciences and Disorders, University of Wisconsin, Madison, WI, United States; ^7^Department of Medical Physics, University of Wisconsin-Madison School of Medicine and Public Health, Madison, WI, United States; ^8^Department of Biostatistics and Medical Informatics, University of Wisconsin-Madison School of Medicine and Public Health, Madison, WI, United States; ^9^Department of Geriatrics, University of Wisconsin-Madison School of Medicine and Public Health, Madison, WI, United States

**Keywords:** cognitively unimpaired, subclinical decline, transitional cognitive decline, mild cognitive impairment, Alzheimer’s disease, validity, biomarkers

## Abstract

While clinically significant cognitive impairment is the key feature of the symptomatic stages of the Alzheimer’s disease (AD) continuum, subtle cognitive decline is now known to occur years before a clinical diagnosis of mild cognitive impairment (MCI) or dementia due to AD is made. The primary aim of this study was to examine criterion validity evidence for an operational definition of “cognitively unimpaired-declining” (CU-D) in the Wisconsin Registry for Alzheimer’s Prevention (WRAP), a longitudinal cohort study following cognition and risk factors from mid-life and on. Cognitive status was determined for each visit using a consensus review process that incorporated internal norms and published norms; a multi-disciplinary panel reviewed cases first to determine whether MCI or dementia was present, and subsequently whether CU-D was present, The CU-D group differed from CU-stable (CU-S) and MCI on concurrent measures of cognition, demonstrating concurrent validity. Participants who changed from CU-S to CU-D at the next study visit demonstrated greater declines than those who stayed CU-S. In addition, those who were CU-D were more likely to progress to MCI or dementia than those who were CU-S (predictive validity). In a subsample with positron emission tomography (PET) imaging, the CU-D group also differed from the CU-S and MCI/Dementia groups on measures of amyloid and tau burden, indicating that biomarker evidence of AD was elevated in those showing sub-clinical (CU-D) decline. Together, the results corroborate other studies showing that cognitive decline begins long before a dementia diagnosis and indicate that operational criteria can detect subclinical decline that may signal AD or other dementia risk.

## Introduction

While clinically significant cognitive impairment tends to occur later in the Alzheimer’s disease (AD) continuum, subtle cognitive decline is now known to occur years before a clinical diagnosis of mild cognitive impairment (MCI) or dementia due to AD is made (Bäckman et al., 2001; Amieva et al., 2008). Recent efforts to describe this preclinical phase of AD have specified cognitive subtypes at increased risk (Sperling et al., 2011). For example, Jack et al. (2018) defined a subset of cognitively unimpaired individuals in the preclinical AD continuum as stage 2 (i.e., transitional cognitive decline) if they evidenced subtle, progressive cognitive decline that did not meet clinical criteria for MCI.

Although current guidelines conceptually define subclinical cognitive decline, researchers and clinicians must ultimately decide how to determine whether subclinical cognitive decline is present. Several groups, including ours, have proposed operational criteria for identifying subtle subclinical cognitive decline (Aisen et al., 2010; Duara et al., 2011; Jessen et al., 2014; Koscik et al., 2014, 2016; Edmonds et al., 2015; Clark et al., 2016). These early criteria typically include the presence of subtle cognitive decline over time and/or lower than expected cognitive performance for sociodemographic expectations, as well as failure to meet criteria for MCI (Albert et al., 2011) or dementia (McKhann et al., 2011). Such criteria may include the option to define subclinical impairment based on the presence of self- or informant-reported subjective cognitive complaints, with or without evidence of subtle cognitive decline on neuropsychological tests (Jack et al., 2018).

To demonstrate that recommended guidelines for subclinical cognitive decline have *criterion validity*, it is important to show that a cognitively unimpaired but declining (CU-D) group, proposed to be at increased risk for progressive cognitive and eventual functional decline, differs from those who are cognitively unimpaired and stable (CU-S) as well as those who meet standard criteria for MCI. If the operational criteria have *concurrent validity*, there should be separation between CU-S, CU-D, and MCI groups in cognitive outcomes sensitive to early AD-related cognitive changes (e.g., absolute scores and within-person change) as well as elevated AD biomarkers among people with CU-D compared to CU-S if the operational criteria are sensitive to concurrent AD pathology. If *predictive validity* is present, faster cognitive decline and/or increased risk of progression to MCI or dementia among those with CU-D would be expected compared to those who are CU-S.

The primary aim of this study was to examine criterion validity evidence for an operational definition of CU-D. Our analyses examined the following hypotheses: (1) CU-S, CU-D, and MCI groups will differ on concurrent objective cognitive performance and subjective reports of functioning; (2) Among those who began CU-S, within-person cognitive declines over subsequent years will vary by cognitive status at the time of follow-up (CU-S, CU-D, MCI/Dementia); (3) Persons identified as CU-D will be at increased risk of progression to a clinical diagnosis (e.g., diagnosis of MCI or dementia) at subsequent visits compared to those who are CU-S; and (4) In the subset who have completed AD-biomarker positron emission tomography (PET) imaging, measures of beta-amyloid plaques and neurofibrillary tangles will vary across cognitive status groups at the most recent cognitive assessment.

## Materials and Methods

### Study Design

Participants were from the Wisconsin Registry for Alzheimer’s Prevention (WRAP), a risk-enriched longitudinal study designed to identify mid-life factors associated with the development of AD (Sager et al., 2005; Johnson et al., 2018). Enrollment of participants began in 2001, with the first follow-up visit (“visit 2”) occurring 2–4 years after the baseline visit and all additional visits occurring at approximate 2-year intervals thereafter; enrollment of new participants, particularly those from underrepresented groups, is ongoing. All study procedures were approved by the University of Wisconsin School of Medicine and Public Health Institutional Review Board and are in concordance with the Declaration of Helsinki.

At the time of selecting WRAP participants who were eligible for these analyses, 1573 people had been enrolled in WRAP and the distribution of most recent number of visits completed was: baseline (i.e., visit 1), *n* = 219; visit 2, *n* = 131; visit 3, *n* = 183; visit 4, *n* = 325; visit 5, *n* = 481; visit 6, *n* = 234 (∼81% overall retention, study baseline mean age = 54 years, 73% with parental history of dementia, and 40% *APOE* ε4 carriers). Participants were excluded from these analyses if they: had completed only the baseline visit (*n* = 219) or had incomplete cognitive data at visit 2 (*n* = 49); reported presence of one or more neurological problems at baseline (*n* = 45; i.e., epilepsy/seizure, multiple sclerosis (MS), and stroke, were not CU-S or CU-D at baseline per review process described in section “Cognitive Status Determination”; *n* = 9), or had non-progressive impairment due to long-standing conditions (e.g., learning disability, *n* = 22), leaving *n* = 1229 meeting initial inclusion criteria. Additional exclusions and corresponding variations in sample sizes are detailed separately below for each hypothesis.

### Study Visit Procedures

At each study visit, participants completed a comprehensive neuropsychological test battery and multiple questionnaires related to lifestyle, health, and subjective self-report of memory functioning. As described in detail by Johnson et al. (2018), the core battery expanded over time to include more measures of memory and executive function and include informant reports of participant functioning. The Clinical Dementia Rating Scale (CDR; Morris, 1997) was added to the protocol in 2012 (initially to the fourth visit and onward) and later used in combination with the Quick Dementia Rating Scale at all study visits (QDRS, Galvin, 2015; Berman et al., 2017) to obtain global ratings (0 = unimpaired, >0 = impaired). The WRAP battery was expanded further in 2014 to include several tests from the CogState computerized cognitive battery (see Hammers et al., 2012; Lim et al., 2013; Racine et al., 2016, for details on CogState). Of the 1229 meeting initial eligibility criteria, 960 (78.1%) had completed at least one CogState assessment (visit 5 = median WRAP study visit of first CogState assessment).

### Cognitive Status Determination

**FIGURE 1:**
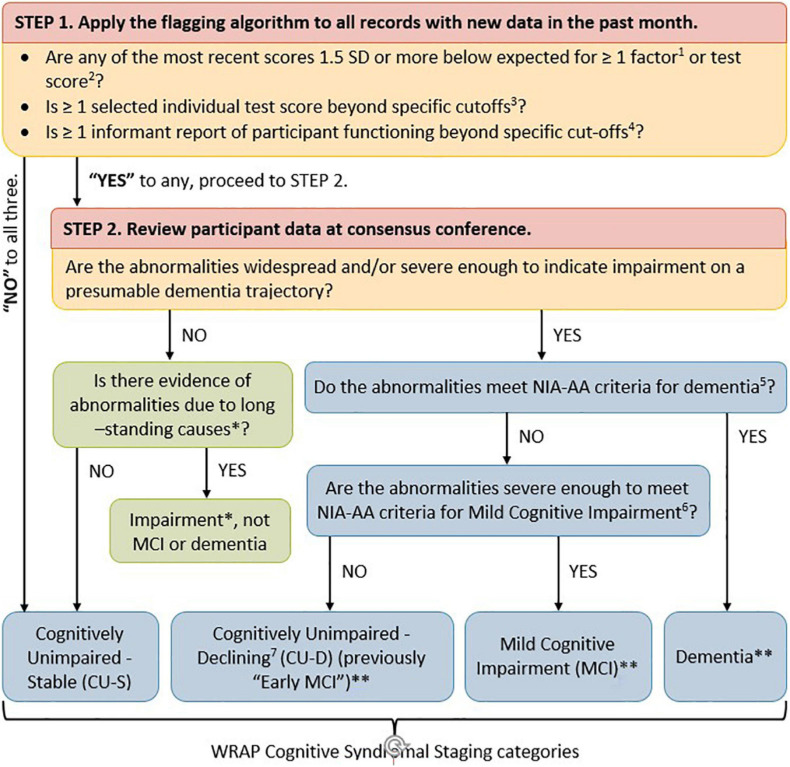
Flow chart of WRAP approach to determining cognitive syndromal staging. Two-tiered review process for determining cognitive status at any given visit. (1) Koscik et al. (2014) describes robust internal norms for factor scores. (2) Clark et al. (2016) describes robust internal norms for individual neuropsychological test scores and how they were combined within theoretical domains. (3) Individual test score cut-offs include: Clock Draw Test ≤ 7; MMSE ≤ 26; AVLT Delayed Recall ≤ 5; and Logical Memory Delayed Recall ≤ 16. (4) Informant report cut-offs include CDR ≥ 0.5; QDRS ≥ 0.5; Lawton IADL < 14; and IQ Code > 52. (5) Jack et al. (2018) [Tables 3, 6 definition of dementia (McKhann et al., 2011)]. (6) Jack et al. (2018) [Tables 3, 6 definition of MCI (Albert et al., 2011)]. (7) As described in Jack et al. (2018; Table 3, and Stage 2 of Table 6). ^∗^Impairment due to something other than probable developing dementia such as long-standing impairment associated with a learning disability or neurological disorder such as epilepsy. ^∗∗^Probable/possible, primary/contributing etiologies assigned. CU-D group was referred to in previous WRAP publications as psychometric MCI and early MCI.

As shown in Figure 1, cognitive status for each visit was determined via a two-tiered review process (Koscik et al., 2016; Johnson et al., 2018). First, visit data were screened by a comprehensive “flagging algorithm” designed to minimize false negatives for subclinical or clinical decline. The flagging algorithm details are shown in Supplementary Table 1. For records that were not flagged, a cognitive status of cognitively unimpaired-stable (CU-S) was assigned. For records that were flagged, the participant’s record was examined during a multi-disciplinary consensus review team which included neuropsychologists, nurse practitioners, geriatricians, psychometricians, and others who review the data. In the review, one team member reviewed the participant’s chart and shared relevant details with the consensus team. After this brief summary, the team also reviewed a snapshot of the participant’s medical history, medications, demographics, longitudinal cognitive performance on a core subset of cognitive measures and longitudinal participant and proxy reports of subjective cognitive and independent function. This review process resulted in one of the following cognitive status assignments for each study visit: cognitively unimpaired-stable (CU-S); cognitively unimpaired-declining (CU-D, i.e., subtle cognitive impairment consistent with a trajectory toward MCI or dementia but not reaching clinical thresholds of impairment)*;* MCI (based on NIA-AA criteria; Albert et al., 2011); dementia (McKhann et al., 2011); and impaired non-MCI (i.e., impairment such as that associated with presence of a learning disability or longstanding brain dysfunction). The CU-D label was assigned when the consensus team determined that cognitive performance was lower than expected based on all available information (including prior performance, or a discrepancy from indicators of crystalized knowledge or from occupational and social histories) but that this decline and current functional status did not reach a threshold of impairment consistent with a diagnosis of MCI or dementia. See Supplementary Table 2 for more details on diagnostic criteria. The consensus review team was blind to biomarker results and performance on the CogState when determining cognitive status leaving the opportunity to consider outcomes that are not circularly linked with cognitive status.

### Study Outcomes

#### Continuous Cognitive Outcomes

Our analyses of continuous outcomes focused on five cognitive composites in the subset of participants who had completed at least one CogState assessment (*N* = 960). Four of the composites were calculated using previously published demographically adjusted *z*-scores for several cognitive tests in the pencil-and-paper portion of the WRAP battery (Clark et al., 2016) including immediate memory, delayed memory, executive function, and a Preclinical Alzheimer Cognitive Composite (PACC; Donohue et al., 2014; Jonaitis et al., 2019). We also created a comparable CogState composite as follows: first, we selected four tests from several we had published on previously (Racine et al., 2016) and which correlated at least modestly (>0.25) with tests contributing to the other four composites. Second, we transformed all scores such that higher indicated better cognitive performance. Third, we computed demographically adjusted *z*-scores for the CogState tests. Each test *z*-score was adjusted for age, gender, and WRAT3 reading standard scores as described previously (Clark et al., 2016); the reading score is considered a marker of literacy/verbal abilities that avoids quality-of-education issues inherent in using years of education (Manly et al., 2003). Composites were calculated by averaging *z*-scores of contributing tests and then converting the average to demographically adjusted standard scores (mean = 100, SD = 15; higher scores indicating better performance). Tests contributing to each composite are shown in Table 1 (top) along with Pearson correlation coefficients among the composite scores (bottom).

**TABLE 1:** Neuropsychological tests contributing to cognitive composites and correlations among composites.

Tests contributing to composites	Immediate learning	Delayed recall	Executive function	PACC3	CogState global
	
	*n* = 958	*n* = 957	*n* = 959	*n* = 952	*n* = 960
**Pencil and paper tests**					
Rey AVLT Total	X	–	–	X	–
Rey AVLT Delayed	–	X	–	–	–
WMS-R Logical Memory-I	X	–	–	–	–
WMS-R Logical Memory-II	–	X	–	X	–
BVMT-R Total	X	–	–	–	–
BVMT-R Delayed	–	X	–	–	–
Stroop Color-Word	–	–	X	–	–
TMT Part B	–	–	X	–	–
WAIS-R Digit Symbol	–	–	X	X	–
CogState tests*					
CPAL	–	–	–	–	X
GML-MPS	–	–	–	–	X
GML-CT	–	–	–	–	X
OCL	–	–	–	–	X

	**Immediate learning**	**Delayed recall**	**Executive function**	**PACC3**	

Pearson correlation matrix (composites only)**					
Delayed recall	0.87				
Executive function	0.29	0.25			
PACC3	0.84	0.77	0.57		
CogState global	0.47	0.47	0.33	0.43	

*The CogState tests were not used in determining cognitive status; prior to adjusting for demographics, CPAL and GML variables were square root transformed and reversed (^∗^−1) so that higher meant better on all four variables.

**p < 0.0001 for all.

*AVLT, Auditory Verbal Learning Task; WMS-R, Wechsler Memory Scale-Revised; BVMT-R, Brief Visuospatial Memory Test-Revised; TMT, Trail Making Test; WAIS-R, Wechsler Adult Intelligence Scale-Revised; CPAL, Continuous Paired Associate Learning; GML-MPS, Groton Maze Learning-Moves Per Second; CT, Chase Test; OCL, One Card Learning.*

#### Subjective Memory Ratings and Informant Reports of Functioning

We analyzed two self-reported memory questions, including “Do you think you have a memory problem” (responses: Yes, no, don’t know; available since study baseline) and “Overall, how would you rate your memory in terms of the kinds of problems that you have” (Likert response ranging from 1 = Major problems to 7 = No problems; available since visit 2; Gilewski et al., 1990). We also analyzed informant reports of participant functioning, including the Instrumental Activities of Daily Living scale (IADL, Lawton and Brody, 1970; range 0–16, <14 indicates possible impaired functioning, Clark et al., 2016) and the Informant Questionnaire of Cognitive Decline in the Elderly (IQCODE; Jorm and Jacomb, 1989; range 16–80, >52 indicates possible impaired function). Informant reports of cognitive functioning were coded as normal or abnormal based on the CDR scale (Morris, 1997) and/or the QDRS, as described in Berman et al. (2017) (>0 indicates abnormal/possible impairment).

#### Progression to Clinical Impairment Status

The primary progression outcome was defined as conversion to MCI or dementia at or after visit 2 and no reversion to non-clinical diagnosis at most recent visit. Secondary progression outcomes included: (1) conversion to MCI or dementia at or after visit 2 (even if someone reverted back to a non-clinical diagnosis at most recent visit), and (2) conversion to MCI or dementia at the subsequent visit from any visit (i.e., next-visit progression).

#### Neuroimaging

Of the 1229 meeting overall inclusion criteria, 262 participants underwent T1-weighted magnetic resonance (MR; GE 3.0 T MR750) and [11C]PIB ([11C]6-OH-BTA-1) (Klunk et al., 2005) amyloid PET imaging at the University of Wisconsin-Madison Waisman Brain Imaging Lab. A subset of 206 also underwent [18F]MK-6240 [6-(Fluoro-18F)-3-(1H-pyrrolo[2,3-c]pyridine-1-yl)isoquinolin-5-amine] tau PET imaging (Hostetler et al., 2016). PET radiopharmaceuticals were synthesized and administered under the Federal Drug Administration Investigational New Drug mechanism. Details regarding PET radioligand synthesis, PET and MRI acquisition protocols, image reconstruction, and post-reconstruction imaging processing have been previously described (Johnson et al., 2014; Betthauser et al., 2019). Image processing was performed using in-house code in MATLAB.

T1-w MRI were tissue-class segmented using the unified segmentation in SPM12^1^. PET regions of interest (ROIs) were generated in subject space by applying the deformation fields defined during segmentation to MNI152 template space atlases [Harvard-Oxford, Desikan et al., 2006; Automated Anatomical Labeling (AAL) Atlas, Tzourio-Mazoyer et al., 2002; MICCAI cerebellum (Landman and Warfield, 2012)] and restricting subject space ROIs to voxels with gray matter probabilities greater than 0.3. PET reference region ROIs were generated by smoothing binary subject space ROIs with a 6 mm isotropic Gaussian kernel (to simulate PET resolution) and keeping resultant voxels with intensity > 0.7.

Positron emission tomography scans were acquired on either a Siemens EXACT HR+ or a Siemens Biograph PET/CT ([11C]PiB: 555 MBq nominal dose, 0–70 min dynamic, 5 × 2-min, 12 × 5-min frames; [18F]MK-6240: 185 or 370 MBq nominal dose, 60–120 or 70–110 min, 5-min frames). Reconstructed PET time series were smoothed, interframe realigned (SPM12), dynamically denoized [HYPR-LR for PiB only (Christian et al., 2010)] and registered to each subject’s T1-w MRI.

Amyloid burden was assessed as a global average distribution volume ratio (DVR; Logan graphical analysis, cerebellum gray matter reference region, k2’ = 0.149 min^–1^ (Lopresti et al., 2005), taken across 8 bi-lateral AAL atlas ROIs (Sprecher et al., 2015). PiB positivity [PiB(+)] was ascertained using global PiB DVR ≥ 1.2 as described previously (Racine et al., 2016). Using recently published amyloid accumulation trajectories and estimated age of reaching PiB(+) (Koscik et al., 2020), we also estimated chronicity of PiB(+) status [i.e., duration PiB(+)] at the cognitive status closest to PET imaging; chronicity was calculated as age at cognitive assessment minus estimated age of reaching the PIB(+) DVR threshold. Thus, PiB+ chronicity estimates the number of years a person has had amyloid above the PiB+ threshold; negative chronicity values indicate estimated time until PiB+.

Tau burden was ascertained from regional [18F]MK-6240 standard uptake value ratios (SUVRs; inferior cerebellum reference region, 70–90 min post-injection) using the anterior parahippocampal gyrus (entorhinal cortex) and hippocampus ROIs from the Harvard-Oxford atlas. These regions were selected as they are posited to be the first regions involved in neurofibrillary tangle deposition and match the sampling regions used in Braak neurofibrillary tangle staging (Braak et al., 2006). We identified thresholds for tau positive within a region as SUVR’s that were >2 SD above mean of the PiB(-) subset; this resulted in cut-offs of SUVR > 1.27 for entorhinal cortex and >1.12 for hippocampus.

### Statistical Analysis

Sample characteristics (e.g., demographics, premorbid IQ estimate, self-reported memory function) were compared across cognitive status groups using tests appropriate to the distribution and number of groups being compared (e.g., *t*-test or ANOVA for normally distributed variables and chi-square or Fisher’s exact for categorical comparisons of two variables; Kruskal–Wallis for comparisons of three groups with non-normal data, etc.). The primary outcome for each hypothesis was tested at alpha = 0.05. When multiple outcomes were of primary interest for a hypothesis, we used the Benjamini–Hochberg false discovery rate (FDR) approach (family-wise error = 0.05; Curran-Everett, 2000). If cognitive status group was significant, post-omnibus pairwise comparisons were conducted. Magnitudes of between-group differences were characterized using Cramer’s V for categorical variables or Cliff’s delta. Cliff’s delta is a robust effect size estimate for continuous and ordinal variables which is less susceptible to outliers and skewness than Hedges’ *g* or Cohen’s *d* and better in circumstances where the homogeneity of variance assumption does not hold (Cliff, 1993). It estimates the probability that a randomly selected observation from one group is larger than a randomly selected observation from another group. Guidelines for negligible, small, medium, and large effect size ranges are listed in footnotes of corresponding tables (Cliff, 1993). Analyses were performed in SAS v9.4, with the exception of Cliff’s delta effect size estimates, which were calculated using the effsize package in R (Torchiano, 2020).

Analyses testing the first hypothesis, that CU-S, CU-D, and MCI groups will differ on concurrent measures of functioning, used data obtained at first CogState assessment (*n* = 960). Analysis of variance (ANOVA), Kruskal–Wallis or Fisher’s exact tests were used (depending on distribution of the outcomes) to determine whether cognitive status group predicted concurrent cognitive composite scores (primary outcomes), and subjective reports of cognition and function (secondary outcomes).

In the subset that was CU-S at first CogState assessment and had a follow-up CogState assessment (*n* = 257), we calculated change as (Standard score at CogState 2 minus Standard score at CogState 1 visit) such that negative scores indicated worsening in cognition. We compared change in composite scores among those who transitioned from CU-S to MCI or CU-D, or remained CU-S, using analysis of covariance (adjusting for years between CogState assessments and baseline score on the same measure to adjust for regression to the mean). In exploratory analyses of this subset, we compared composites from the first CogState visit across cognitive status groups at the second CogState to examine whether cognitive differences were apparent already at the first CogState visit when all in the subset were still considered CU-S. In sensitivity analyses of continuous composites and change in composites, we ran Kruskal–Wallis tests; for models that had included covariates, the Kruskal–Wallis tests were performed on the residuals from the covariate(s)-only models.

We used the larger set of *n* = 1229 participants and logistic regression to test our third hypothesis that CU-D status was associated with increased risk (compared to CU-S) of subsequently being diagnosed with cognitive impairment (MCI or dementia; adjusting for sex, baseline age, WRAT3 reading and years of follow-up). Given the low prevalence of MCI in our sample, prior to testing hypothesis 3, we conducted preliminary power calculations to ensure we had adequate statistical power to detect meaningful differences in progression rates between CU-S and CU-D groups. Based on a whole sample progression rate of 3–4%, we estimated that we had over 80% power (two-tailed alpha = 0.05) to detect plausible differences in progression rates between CU-S and CU-D such as 2.5% vs. 10.1%; 3% vs. 10.8%; and 3.5% vs. 11.5%. In descriptive analyses, we also characterized visit-to-visit stability of CU-D and MCI by reporting proportions and confidence intervals for proportions of people reverting at next visit to a less impaired status from CU-D and MCI, respectively.

Using the cognitive status closest to the most recent PET imaging in the subset with amyloid PET (*n* = 262) and tau PET (*n* = 209) to test hypothesis 4, we compared Global PiB DVR (amyloid plaque accumulation), and estimated PiB+ chronicity at the time of the cognitive assessment (Koscik et al., 2020) across CU-S, CU-D and MCI groups using the Kruskal–Wallis test to compare the distributions (with follow-up pairwise 2-sample Wilcoxon if the Kruskal–Wallis test was significant). We performed the same analyses on entorhinal cortex and hippocampus MK-6240 SUVR’s (neurofibrillary tangle accumulation). We used Fisher’s exact tests to examine whether the proportion passing thresholds for elevated PiB or elevated entorhinal or hippocampal MK-6240 SUVR differed across cognitive status groups (see section “Neuroimaging” for thresholds).

## Results

### CU-S, CU-D, and MCI Group Differences on Concurrent Objective Cognitive Performance and Subjective Reports of Functioning

Nine hundred and sixty participants had at least one CogState cognitive composite and were thus eligible for inclusion in Aim 1 analyses. We focused on this subset for concurrent validity evidence to minimize circularity (CogState scores are not reviewed in any part of the consensus review process). In this subsample, the CU-S and MCI groups had more non-Hispanic Caucasians than the CU-D group; the CU-S group was younger than both other groups; and the CU-S group had a higher proportion of women than the MCI group (details and additional sample characteristics in Table 2). The CogState composite correlated moderately with the four pencil-and-paper based cognitive composites (Pearson rho range = 0.33–0.47; Table 1).

**TABLE 2:** Sample characteristics by cognitive status at first CogState assessment for subset used in analyses using CogState data.

	Cognitive status at 1st CogState				
	
	CU-S	CU-D	MCI	*p*-value**	Pairwise info
Sample characteristics	*n* = 816	*n* = 123	*n* = 21		
Age at first CogState assessment, mean (SD)	64.0 (6.4)	65.9 (6.4)	68.3 (4.6)	0.0002	CU-S and MCI; CU-D and MCI differ
Years of education (max 20), median (Q1–Q3)	16 (14–18)	16 (14–18)	14 (13–16)	0.12	
WRAT3 reading recognition, mean (SD)	106.3 (8.9)	105.8 (8.7)	104.9 (12.2)	0.63	
Female, *n* (%)	568 (69.6)	75 (61.0)	10 (47.6)	0.022	CU-S vs. MCI
*APOE* ε4 carrier, *n* (%)	309 (37.9)	51 (41.5)	10 (47.6)	0.49	
Non-Hispanic Caucasian, *n* (%)*	788 (96.6)	107 (87.0)	20 (95.2)	0.0001	CU-D < CU-S

*Among the *n* = 28 in CU-S group who are not non-Hispanic Caucasian, 13 (21.4%) were African American compared with 12 of 16 (75%) in the CU-D group and 0 of 1 in the MCI group.

**p-values are from analysis of variance for rows reporting mean (SD), Kruskal–Wallis for rows reporting medians, and chi-square or Fisher’s exact for rows reporting *n* (%).

*CU-S, cognitively unimpaired-stable; CU-D, cognitively unimpaired-declining; MCI, mild cognitive impairment; WRAT, wide range achievement test; APOE, apolipoprotein.*

**FIGURE 2:**
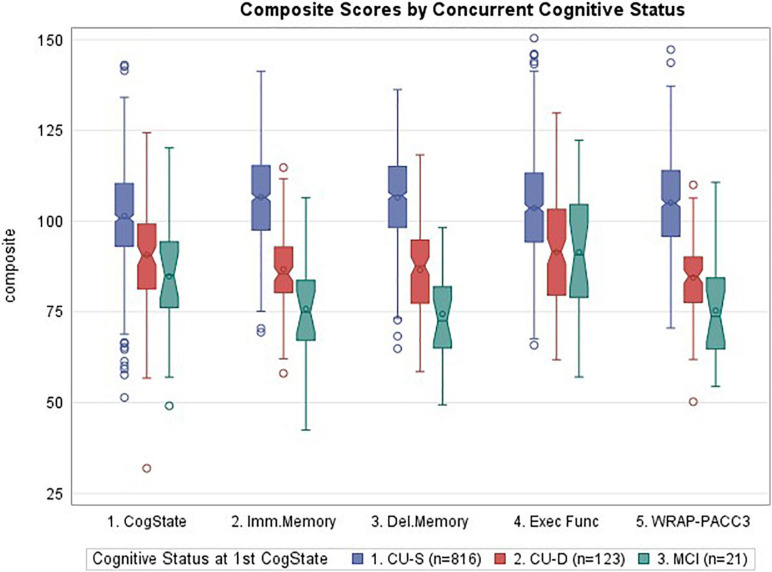
Notched boxplots of cognitive composites by concurrent cognitive status, where notches represent the median ± 1.58*(interquartile range/square root of *n*). CogState, CogState composite; Imm.Memory, immediate memory composite; Del.Memory, delayed memory composite; Exec Func, executive function composite; WRAP-PACC3, WRAP’s version of the preclinical Alzheimer’s cognitive composite (see Table 1 for tests contributing to each composite). Values represent demographically adjusted standard scores [mean (SD) = 100 (15)]. *P* < 0.0001 for all five composites; pairwise differences and effect sizes indicated in Table 3.

Figure 2 depicts a consistent pattern of lower demographically adjusted composite scores across the concurrent CU-D and MCI/Dementia groups compared to CU-S MCI/Dementia groups; this predictor was significant for all five cognitive composites (ANOVA *p* < 0.0001 for each). Means (SD) for each composite are shown by cognitive status group in the top portion of Table 3 along with pairwise Cliff’s delta effect sizes. Follow-up pairwise comparisons showed that all pairs differed significantly for the Immediate Memory, Delayed Memory and PACC3 composites. For the CogState and Executive Function Composites, only the CU-D and MCI/Dementia comparison did not differ significantly (*p* = 0.07 for Cogstate, and *p* = 0.97 for Executive Function). Effect sizes for CU-S vs. CU-D ranged from medium (CogState and Executive Function) to large (the other three composites). Effect sizes for CU-D vs. MCI ranged from negligible (Executive Function) to large (Delayed Memory). Model diagnostics suggested some potential influential observations, so we conducted sensitivity analyses using non-parametric Kruskal–Wallis tests; significance patterns were unchanged and effect sizes were similar.

**TABLE 3:** Concurrent validity evidence (subset with CogState).

	1st CogState Cog Status				Post-omnibus pairwise comparisons and Cliff’s delta		
		
Cognitive composites	CU-S *n* = 816	CU-D *n* = 123	MCI *n* = 21	Omnibus *p*-value*	CU-S vs. CU-D *p*-value, Cliff’s delta	CU-S vs. MCI *p*-value, Cliff’s delta	CU-D vs. MCI *p*-value, Cliff’s delta
CogState (*n* = 960), mean (SD)	101.4 (13.5)	90.7 (14.4)	84.8 (17.3)	<0.0001	<0.0001, 0.42	<0.0001, 0.57	0.069, 0.22
Immediate memory (*n* = 958), mean (SD)	106.6 (12.2)	86.7 (11.1)	75.8 (14.1)	<0.0001	<0.0001, 0.77	<0.0001, 0.89	0.0001, 0.50
Delayed memory (*n* = 957), mean (SD)	106.5 (11.9)	86.5 (13.0)	74.4 (12.9)	<0.0001	<0.0001, 0.74	<0.0001, 0.92	<0.0001, 0.49
Executive function (*n* = 959), mean (SD)	103.6 (14.0)	91.5 (15.6)	91.4 (17.3)	<0.0001	<0.0001, 0.42	0.0001, 0.40	0.97, 0.009
PACC3 (*n* = 952), mean (SD)	105.0 (12.7)	84.5 (11.2)	75.3 (14.7)	<0.0001	<0.0001, 0.77	<0.0001, 0.85	0.0028, 0.42

**Subjective reports of functioning**	**CU-S**	**CU-D**	**MCI**	Omnibus *p*-value*	**CU-S vs. CU-D *p*-value, Cramer’s V**	**CU-S vs. MCI *p*-value, Cramer’s V**	**CU-D vs. MCI *p*-value, Cramer’s V**

Self-report of memory problems at time of first CogState				0.0043	0.015, 0.095	0.023, 0.091	0.48, 0.10
Yes, *n* (%)	139 (17.1)	29 (23.6)	7 (33.3)				
Don’t know, *n* (%)	141 (17.4)	30 (24.4)	6 (28.6)				
No, *n* (%)	532 (65.5)	64 (52.0)	8 (38.1)				
	
					**CU-S vs. CU-D *p*-value, effect size**	**CU-S vs. MCI *p*-value, effect size**	**CU-D vs. MCI *p*-value, effect size**
	
Likert scale self-memory rating, 1 is worst, 7 is best; median [Q1–Q3]	5 [4–6]	5 [4–6]	5 [4–5]	0.056	NA	NA	NA
IADL, 16 is best, median [Q1–Q3]	16 [16–16]	16 [16–16]	16 [16–16]	0.0034	0.002, 0.17	0.08, 0.19	0.81, 0.19
IQ-Code, 48 is no change, median [Q1–Q3]	48 [48–48]	48 [48–49]	49 [48–51]	0.0007	0.045, 0.013	0.007, 0.25	0.054, 0.014
QDRS/CDR > 0, *n* (%) [total *n* = 792]	21 (3.1%)	11 (12.4%)	8 (42.1%)	<0.0001	0.0004, 0.15	<0.0001, 0.32	0.005, 0.30

*Omnibus *p*-values and follow-up pairwise comparison *p*-values from ANOVA for mean (SD), Kruskal–Wallis for median [Q1–Q3] and chi-square/Fisher’s exact test for *n* (%).

*Effect sizes are either Cliff’s *d* [for mean (SD) or median [Q1–Q3] variables] or Cramer’s V [for *n* (%) variables]. Cliff’s *d*’s obtained in R using ‘cliff.delta’ function (absolute value reported). Magnitude can be assessed using the thresholds provided in Romano et al. (2006), i.e., | *d*| < 0.147 “negligible,” | *d*| < 0.33 “small,” | *d*| < 0.474 “medium,” otherwise “large”; Cramer’s V effect sizes are considered low/weak at ∼0.1.*

*All Cog composites sig using BH corrected *p*-values. Secondary outcomes: least sig (memrate) compared to *p* = 0.05. NS. Memprobs compared to.05/4 of.0125. That test and others in set are significant.*

Chi-square tests showed that cognitive status at first CogState visit was associated with the self-reported memory problem item, “Do you think you have a memory problem” (Table 3); follow-up pairwise analyses indicate that fewer participants endorsed *no* memory problems in the CU-D and MCI groups than in the CU-S group, although Cramer’s V values indicated these relationships represented weak effect sizes. The Kruskal–Wallis test of the seven-point Likert scale item, “Overall, how would you rate your memory in terms of the kinds of problems that you have” showed no significant differences across the three groups (*p* = 0.056; Table 3). Despite ratings generally showing little functional impairment in our sample, IADL and IQCODE ratings differed across concurrent cognitive status groups; follow-up comparisons showing significant IADL differences between the CU-S and CU-D group and significant IQCode differences between CU-S and each of the other groups (Table 3). CDR ratings also differed across all groups, with 3%, 12%, and 42% of the CU-S, CU-D, and MCI/Dementia group, respectively, having a rating greater than 0.

### Examining Within-Person Cognitive Declines Over Subsequent Years From CU-S at First CogState to CU-S, CU-D, or MCI/Dementia at Second CogState

In the subset that was CU-S at first CogState and who also had a second CogState assessment (*n* = 257), changes in standard scores from first to second CogState differed across cognitive status groups at second CogState for all composites (largest *p* = 0.0065, Executive Function; see Table 4 for descriptive statistics). Pairwise follow-up comparisons showed that all cognitive status pairs differed in change for the PACC and memory composites. For the CogState composites, the CU-S vs. MCI/Dementia comparison was significant and CU-S vs. CU-D differences were marginal/weak (*p* = 0.051). Similarly, for Executive function CU-S and MCI differed significantly while CU-D vs. MCI was marginal/weak (*p* = 0.056). Effect sizes for CU-S vs. CU-D ranged from negligible (Executive function) to large (the Memory and PACC composites). Effect sizes for CU-S vs. MCI were all large. Effect sizes for CU-D vs. MCI were small for the CogState composite and large for the other four composites. In sensitivity analyses using non-parametric Kruskal–Wallis tests on the residuals from models that adjusted only for score at CogState 1 and years between CogState 1 and 2, significance patterns were unchanged except for two contrasts: CogState composite, CU-S vs. CU-D, Kruskal–Wallis *p*-value = 0.035; Delayed Memory, CU-D vs. MCI, Kruskal–Wallis *p*-value = 0.077).

**TABLE 4:** Cognitive composites and change in subset that had two CogState assessments.

	By last cognitive status				Follow-up pairwise *p*-values, Cliff’s delta effect sizes		
			
	CU-S *n* = 235	CU-D *n* = 16	MCI/Dementia *n* = 6	*p*-value	CU-S vs. CU-D	CU-S vs. MCI/Dementia	CU-D vs. MCI/Dementia
Age at CogState 1, mean (SD)	63.5 (6.4)	64.7 (7.0)	67.4 (5.0)	0.26	NA	NA	NA
Years between CogState 1 and 2, mean (SD)	2.4 (0.3)	2.5 (0.4)	2.5 (0.4)	0.81	NA	NA	NA
**Change in composites from CU-S at CogState 1 to CU-S, CU-D or MCI at CogState 2**							
CogState composite, lsmean (SE)	3.3 (0.8)	−2.7 (2.9)	−10.7 (4.8)	0.0038	0.051 (KW *p* = 0.035), 0.31	0.0046, 0.52	0.16, 0.33
Immediate memory, lsmean (SE)	1.7 (0.6)	−11.9 (2.3)	−29.5 (3.6)	<0.0001	<0.0001, 0.68	<0.0001, 0.93	<0.0001, 0.60
Delayed memory, lsmean (SE)	2.4 (0.6)	−10.9 (2.2)	−31.0 (3.6)	<0.0001	<0.0001, 0.64	<0.0001, 0.84	<0.0001 (KW *p* = 0.077), 0.50
Executive function, lsmean (SE)	1.4 (0.5)	−0.91 (1.8)	−7.4 (2.9)	0.0065	0.21, 0.12	0.003, 0.70	0.056, 0.50
PACC3, lsmean (SE)	1.9 (0.5)	−10.2 (2.0)	−26.4 (3.3)	<0.0001	<0.0001, 0.63	<0.0001, 0.97	<0.0001, 0.73
**Composites at CogState 1 by status at CogState 2**							
CogState, lsmean (SE)	102.5 (0.9)	95.5 (3.4)	89.7 (5.6)	0.015*	0.051, 0.25	0.026 (KW *p* = 0.055), 0.46	0.38, 0.10
Immediate memory, lsmean (SE)	107.8 (0.8)	97.0 (3.0)	100.0 (4.9)	0.0009	0.0005, 0.44	0.12, 0.40	0.60, 0.17
Delayed memory, lsmean (SE)	107.3 (0.7)	99.4 (2.9)	89.7 (3.0)	<0.0001	0.008, 0.37	0.0002, 0.69	0.078, 0.42
Executive function, lsmean (SE)	104.4 (0.9)	101.6 (3.3)	93.9 (5.0)	0.13	NA, 0.09	NA, 0.53	NA, 0.27
PACC3, lsmean (SE)	106.3 (0.8)	98.8 (3.0)	93.9 (5.0)	0.0039	0.018, 0.31	0.014, 0.64	0.39, 0.38

For change in composites, lsmeans are adjusted for baseline score on that composite and years between CogState 1 and 2; For the CogState 1 analysis of scores by CogState 2 cognitive status (same subset), lsmeans adjust for time between CogState 1 and 2.

*(KW *p* = 0.044. marginal by FDR correction).

*Cliff’s *d*’s obtained in R using ‘cliff.delta’ function on the covariate-adjusted residuals of the change in standard scores for each composite (upper half of table) and standard scores for each composite at CogState 1 (lower half-subjective reports of functioning). Magnitude can be assessed using the thresholds provided in Romano et al. (2006), i.e., | *d*| < 0.147 “negligible,” | *d*| < 0.33 “small,” | *d*| < 0.474 “medium,” otherwise “large.”*

In exploratory analyses of this subset that was CU-S at first CogState visit, we also examined whether subtle cognitive differences were evident at the first CogState among those who were CU-D or MCI/Dementia at their second CogState visit. As shown in the bottom of Table 4, average performance in each of the three groups was clearly in a non-impaired range at the time of the first CogState assessment (group standard score averages ranging from 89.7 to 107.8). After adjusting for years between the first and second CogState assessments, all composites except the Executive function composite showed significant group effects. Follow-up pairwise comparisons showed that for those that were CU-S vs. CU-D at second CogState, scores at CogState 1 differed for both memory composites and the PACC composite with effect sizes ranging from small to medium; differences on the CogState were marginal/weak (*p* = 0.051, small effect size).

### Examining Whether Persons Identified as CU-D Were at Increased Risk of Progression to Clinical Impairment Compared to Those Who Were CU-S

The *n* = 1229 who met initial eligibility criteria were used to test the hypothesis that CU-D at baseline was associated with higher risk of progression to MCI compared with CU-S at baseline (hypothesis 3). Sample characteristics are presented in Table 5, by CU-S vs. CU-D baseline groups. The CU-D at baseline group (*n* = 119) was 1.8 years older on average and had more males and more participants from underrepresented groups. In addition, the CU-D group had lower demographically adjusted *z*-scores for AVLT total, AVLT delay, and Trails B (tests used in the composites that were available at baseline). Both cognitive status groups had completed a median of five study visits, corresponding to a mean (SD) of 10.2 (3.0) years of follow-up.

**TABLE 5:** Sample characteristics in larger sample used to examine the progression hypothesis.

	Baseline cognitive status (*n* = 1229)		
	
	CU-S	CU-D	*p*-value**
Demographics	*n* = 1110	*n* = 119	
Age, mean (SD)	**54.0 (6.6)**	**55.8 (5.9)**	**0.006**
Years of education (max 20), median (Q1–Q3)	16 (14–18)	16 (14–18)	0.25
Literacy/VIQ, mean (SD) WRAT3 Reading standard score	106.1 (9.1)	105.1 (9.5)	0.29
Female, *n* (%)	**792 (71.4)**	**60 (50.4)**	**<0.0001**
*APOE* ε4 carrier, *n* (%)	425 (38.3)	46 (38.7)	0.94
Race/ethnicity = non-Hispanic Caucasian, *n* (%)*	**1052 (94.8)**	**103 (86.6)**	**0.002**
Self-report of memory problems			0.34
Yes, *n* (%)	264 (23.9)	32 (26.9)	
Don’t know, *n* (%)	208 (18.8)	27 (22.7)	
No, *n* (%)	634 (57.3)	60 (50.4)	
Baseline IICV*, adjusted mean (SE)	**0.69 (0.012)**	**1.18 (0.033)**	**<0.0001**
AVLT Total, adjusted mean (SE)	**0.02 (0.027)**	−**1.22 (0.076)**	**<0.0001**
AVLT Delay, adjusted mean (SE)	**0.04 (0.027)**	−**1.30 (0.077)**	**<0.0001**
Trails B, adjusted mean (SE)	**0.07 (0.029)**	−**0.95 (0.082)**	**<0.0001**

*Among the *n* = 58 in CU-S group who are not non-Hispanic Caucasian, 30 (51.7%) were African American compared with 11 of 16 (68.8%) in the CU-D group.

**p-values are from *t*-tests for rows reporting mean (SD), Mann–Whitney *U* or Kruskal–Wallis for rows reporting medians, and chi-square or Fisher’s exact for rows reporting *n* (%).

*CU-S, cognitively unimpaired-stable; CU-D, cognitively unimpaired-declining; MCI, mild cognitive impairment; IICV, intraindividual cognitive variability calculated as the standard deviation of the baseline *z*-scores of WRAT reading, AVLT Total, AVLT delayed recall, and Trails B.*

**FIGURE 3:**
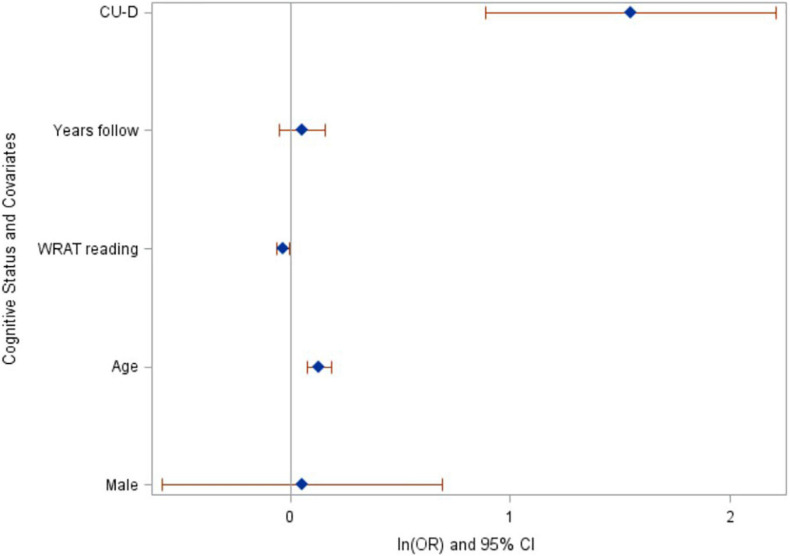
Baseline cognitive status of CU-D predicts increased risk of progressing to MCI/Dementia at last assessment. Ln(odds ratios) and 95% CI’s from logistic regression model. CU-D, cognitive unimpaired-declining.

Forty-eight participants (3.9%) had progressed to MCI or dementia at their most recent cognitive assessment (*n* = 42 progressed to MCI, *n* = 6 progressed to dementia). Among the 1110 who were CU-S at baseline, *n* (%) = 32 (2.9%) progressed to a clinical status (95% CI = 1.9 to 3.9%) compared with 16 of 119 (13.5%) who were CU-D at baseline (95% CI = 7.4 to 19.6%). In logistic regression analyses, CU-D at baseline was associated with higher risk of progressing to a clinical status at most recent assessment (*p* < 0.0001, after adjusting for baseline age, sex, literacy and follow-up years; see Figure 3 for model Odds Ratios and 95% CIs). These results suggest CU-D has predictive validity for MCI.

**FIGURE 4:**
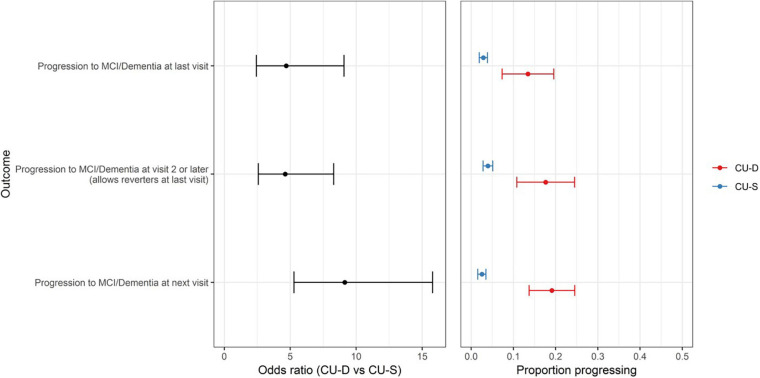
CU-D increases risk of later clinical status in secondary progression outcomes. **(Left)** Odds ratios and 95% CI’s from logistic regression models showing risk of progression for primary outcome (top row) and secondary outcomes (MCI/Dementia at visit 2 or later; and MCI/Dementia at “next visit” from CU-S or CU-D at previous visit). CU-S, cognitively unimpaired-stable; CU-D, cognitive unimpaired-declining. **(Right)** 95% confidence interval for proportion progressing to MCI/Dementia, by CU-D and CU-S.

Patterns were consistent in our secondary outcomes. Sixty-five participants had progressed to a clinical status at any visit after baseline (allowing reversion to non-clinical status at most recent visit). In logistic regression analyses, CU-D at baseline was associated with higher risk of progressing to a clinical status any time after baseline [OR (95% CI) = 4.6 (2.6–8.3); *p* < 0.0001]. Similarly, CU-D predicted greater risk than CU-S of progressing to a clinical status *at the next visit* [OR (95% CI) = 9.1 (5.3–15.8); *p* < 0.0001; 2.5% of CU-S progressed vs. 19.1% of CU-D at next visit; Figure 4].

#### Reversion to Less Impaired Cognitive Statuses

In exploratory analyses we calculated the 95% CI for proportion reverting to less impaired status as a descriptor of the stability of CU-D and MCI. Of the 558 CU-D visits with a follow-up status (i.e., allowing repeat CU-D statuses within person across visits), 269 (48.2%) reverted to CU-S at the next visit (95% CI = 44%–52% reversion). Similarly, 43 of 77 MCI visits had follow-up statuses; in this subset, 13 (30.2%) reverted to CU-D (CI = 17.7–46.3) and 9 (20.9%) reverted to CU-S (CI = 10.6–36.5) at the next visit.

### Examining Whether AD-Biomarker PET Imaging, Measures of Beta-Amyloid Plaques and Neurofibrillary Tangles Vary Across Cognitive Status Groups

**FIGURE 5:**
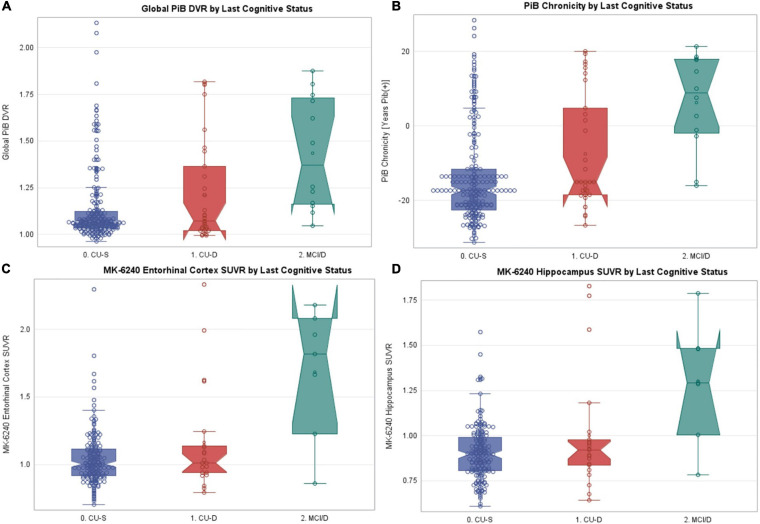
PET PiB, PiB chronicity and MK-6240 values by last cognitive status. Notched boxplots of amyloid and tau PET data, where notches represent the median ± 1.58*(interquartile range/square root of *n*). **(A)** Most recent global PET PiB DVR value by most recent cognitive status. **(B)** Estimated PiB chronicity at time of most recent cognitive status. **(C)** MK-6240 entorhinal cortex SUVR by most recent cognitive status. **(D)** MK-6240 hippocampal SUVR by most recent cognitive status. CU-S, cognitively unimpaired-stable; CU-D, cognitive unimpaired-declining; MCI/D, MCI or dementia. N’s within the CU-S, CU-D, and MCI/Dementia groups were, respectively 219, 31, 12 for PET PiB, and 181, 21, 7 for MK-6240.

Sample characteristics and PiB and MK-6240 summary data for the subset with PiB PET (*n* = 262) or MK-6240 PET (*n* = 209) are found in Table 6 by cognitive status at most recent neuropsychological assessment. Sample characteristics of the PiB subset were similar to the larger sets. Global PiB DVR differed between cognitive status groups (Kruskal–Wallis *p* = 0.0003); follow-up pairwise comparisons showed that both CU-S and CU-D differed from MCI (large Cliff’s delta effect sizes), though not from each other (negligible effect size). Parallel analyses of PiB chronicity at time of cognitive assessment differed across all pairs (Table 6; CU-S vs. CU-D small effect size; large for other pairs) with higher average years of PiB(+) duration as level of impairment worsened. Notched boxplots of Global PiB DVR and PiB chronicity are shown in Figures 5A,B, respectively (top row). Fisher’s exact test indicated that the proportion PiB(+) differed across cognitive status groups. Follow-up pairwise comparisons indicated that the proportion PiB(+) differed between CU-S and CU-D (*p* = 0.033) and CU-S and MCI (*p* = 0.0005), but not CU-D vs. MCI (*p* = 0.092). The proportion PiB(+) was highest in the MCI group (66.7%, 95% CI: 35.4% to 88.7%). Approximately 35.5% in the CU-D group were PiB(+), yielding a 95% CI of 19.8 to 54.6% compared with approximately 18.3% PiB(+) in the CU-S group (95% CI of 13.5% to 24.2%).

**TABLE 6:** PiB and MK-6240 PET subset by most recent cognitive status.

	Most recent cognitive status (*n* = 262*)						
		
	CU-S	CU-D	MCI/Dementia	*p*-value**	Pairwise diffs:		
**Sample characteristics**	**219 (83.6%)**	**31 (11.8%)**	**12 (4.6%)**				
Most recent cognitive status age, mean (SD)	66.4 (6.4)	69.2 (4.8)	70.8 (5.7)	0.006	CU-S vs. CU-D and MCI		
Literacy/WRAT3, mean (SD)	107.2 (8.7)	106.5 (9.7)	108.8 (7.5)	0.76			
Years of education (max 20), median [Q1–Q3]	16 [14–18]	17 [14–18]	16.5 [13–17.5]	0.54			
Female, *n* (%)	155 (70.8%)	15 (48.4%)	8 (66.7%)	**0.049**	CU-S vs. CU-D		
*APOE* ε4 carrier, *n* (%)	84 (38.4%)	14 (45.2%)	7 (58.3%)	0.30			
Race/ethnicity = non-Hispanic Caucasian, *n* (%)*	210 (95.9)	27 (87.1)	11 (91.7)				
Memory rating (1 = worst, 7 = best), median [Q1–Q3]	5 [5–6]	5 [4–5.5]	4 [3–4]	**0.0006**	CU-S vs. MCI; CU-D vs. MCI (0.05 < *p* < 0.1 for CU-S vs. CU-D)		
Concurrent QDRS/CDR > 0, *n* (%) [total *n* = 143]	3 (2.6%)	3 (16.7%)	6 (66.7%)	**<0.0001**	All pairs		
**PET scan information**					**Follow-up pairwise *p*-values, Cliff’s delta effect sizes**		
PiB scan age – cognitive status age, mean (SD)	−0.22 (2.5)	−0.53 (3.0)	0.27 (1.58)	0.63	**CU-S vs. CU-D**	**CU-S vs. MCI/Dementia**	**CU-D vs. MCI/Dementia**
Global PiB DVR, median [Q1–Q3]	1.06 [1.03–1.12]	1.07 [1.02–1.36]	1.37 [1.16–1.73]	**0.0003**	0.56, 0.065	**<0.0001, 0.70**	**0.0097, 0.52**
PiB chronicity at last NP, median [Q1–Q3]	−17.3 [−22.6, −11.6]	−15.0 [−18.6, 4.80]	8.9 [−1.9, 18.0]	**<0.0001**	**0.014, 0.27**	**<0.0001, 0.73**	**0.011, 0.51**
Elevated PiB (≥1.2), *n* (%)	40 (18.3%)	11 (35.5%)	8 (66.7%)	**<0.0001**	**0.033, 0.14**	**0.0005, 0.26**	0.092, 0.28
MK-6240 scan subset (*n* = 209*)	**CU-S**	**CU-D**	**MCI**	*p*-value**			
	181 (86.5%)	21 (10.3%)	7 (3.3%)				
MK-6240 scan age, mean (SD)	67.2 (6.4)	68.9 (4.3)	73.2 (4.0)	0.024			
MK-6240 scan age – Cog Status age, mean (SD)	0.61 (1.21)	0.59 (0.87)	0.86 (1.05)	0.85	**CU-S vs. CU-D**	**CU-S vs. MCI/Dementia**	**CU-D vs. MCI/Dementia**
MK-6240 entorhinal SUVR, median [Q1–Q3]	1.00 [0.92–1.11]	1.01 [0.94–1.14]	1.82 [1.22–2.08]	**0.0062**	0.41, 0.40	**0.0018, 0.82**	**0.024, 0.75**
MK-6240 hippocampus SUVR, median [Q1–Q3]	0.90 [0.81–0.99]	0.92 [0.84–0.98]	1.29 [1.00–1.48]	**0.0085**	0.48, 0.39	**0.0021, 0.82**	**0.047, 0.71**
Elevated MK-6240 entorhinal SUVR, *n* (%)	15 (8.1%)	4 (18.2%)	5 (71.4%)	**<0.0001**	0.12, 0.11	**0.0002, 0.39**	**0.020, 0.49**
Elevated MK-6240 hippocampus SUVR, *n* (%)	10 (5.4%)	4 (18.2%)	5 (71.4%)	**<0.0001**	**0.044, 0.16**	**<0.0001, 0.46**	**0.020, 0.49**

*Among the *n* = 9 in CU-S group who are not non-Hispanic Caucasian, 4 (%) were African American compared with 3 of 4 (75%) in the CU-D group and 1 of 1 in the MCI group.

**p-Values are from *t*-tests for rows reporting mean (SD), Kruskal–Wallis for median [Q1–Q3] and chi-square/Fisher’s exact test for *n* (%). Means adjusted for age at scan.

*CU-S, cognitively unimpaired-stable; CU-D, cognitively unimpaired-declining; MCI, mild cognitive impairment.*

In parallel analyses of the subset with MK-6240 SUVR data (*n* = 209), Kruskal–Wallis tests indicated that both entorhinal cortex and hippocampal SUVR differed by cognitive status (Table 6 and Figures 5C,D, respectively). In follow-up pairwise comparisons, CU-S and CU-D MK-6240 SUVR levels did not differ in either region (*p* = 0.41, entorhinal cortex; *p* = 0.48, hippocampus; medium Cliff’s d effect sizes); the CU-S and MCI groups differed across both regions (*p* = 0.0018, entorhinal cortex; *p* = 0.0021, hippocampus; large effect sizes); and CU-D and MCI differed on both regions (*p* = 0.024, entorhinal cortex; *p* = 0.047, hippocampus; large effect sizes). Fisher’s exact test indicated that the proportion with elevated entorhinal SUVR or elevated hippocampus SUVR differed by cognitive statuses (*p* < 0.0001 for both). For both ROI’s, the proportion with elevated SUVR was higher in the MCI group compared to CU-S (*p* = 0.0002, entorhinal cortex; *p* < 0.0001, hippocampus). The proportions differed between CU-S and CU-D for hippocampus (*p* = 0.044) but not the entorhinal cortex (*p* = 0.12). Despite the small sample sizes, differences in proportions with elevated MK-6240 SUVR in the 17 with CU-D vs. 7 with MCI were significant (*p* = 0.02, entorhinal cortex; *p* = 0.02, hippocampus; Table 6).

## Discussion

We presented a two-tiered consensus diagnosis approach to determining cognitive status in the WRAP study. Through the use of published norms and internal demographically adjusted norms, a flagging algorithm identified people with potential clinical or subclinical deficits. The multidisciplinary team reviewed those identified by the algorithm and determined whether the performance was consistent with traditional categories of MCI or dementia. For those who did not meet clinical criteria, the team determined whether the flagged deficits were severe enough to warrant a subclinical category called cognitively unimpaired-declining (CU-D). We then examined the concurrent and predictive criterion-related validity of our CU-D category. Four major findings resulted, and each is discussed below. Overall, evidence supports the idea that subclinical cognitive decline, a stage of disease progression important in the examination of preclinical AD, can be detected and that for a subset of those with such deficits, signs of AD-related brain pathology are also evident.

### Finding 1: CU-S, CU-D and MCI Cognitive Status Groups Differed in Concurrent Objective Cognitive Performance and Subjective Reports of Functioning

Concurrent cognitive status was a significant predictor for all cognitive composite metrics with a consistent pattern of lower average demographically adjusted standard scores from CU-S to CU-D to MCI. Importantly, the CU-S average CogState score was 10 points higher than the CU-D group and the CU-D group average was six points higher than the MCI group [on a scale with mean (SD) 100 (15)]; since CogState scores were not used in consensus conference decisions and correlations between this composite and the other composites was modest (0.33–0.47), this provided non-circular evidence of separation among cognitive status groups. Between-group effect sizes for the CogState comparisons ranged from small (CU-D vs. MCI) to large (CU-S vs. MCI). Not surprisingly, between group effect sizes were generally larger for the composites calculated from tests used in consensus review, with the memory and PACC composites all showing medium to large effect sizes. Although the tests comprising the Executive function composite are reviewed during consensus conferences, this composite correlated most weakly with the other four and showed the smallest between-group effect sizes.

Concurrent between-cognitive-group differences in informant ratings of functioning were consistent with the CogState results, showing little to no functional difficulties in CU-S and increasing slightly across the CU-D and MCI groups. Subjective self-report of memory problems were inconsistent with no overall group difference on the Likert index but a group difference on the single item with fewer participants endorsing no memory problems in CU-D and MCI than CU-S. A previous study (Edmonds et al., 2018) showed similar patterns; in that study, self-reported subjective cognitive complaints became increasingly discordant with objective cognitive status as objective cognitive impairment became more pronounced.

Overall, concurrent validity for the operationalization of CU-D is provided by the consistency across these objective and subjective measures. The stair-step decreases from CU-S to CU-D to MCI are consistent with the suggestion that CU-D is a preclinical cognitive condition antecedent to a clinical diagnosis of MCI.

### Finding 2: Among Those Who Began CU-S, Within-Person Cognitive Declines at Next Visit Varied by Cognitive Status at Follow-Up

To reduce circularity with consensus status determination, our analyses of within-person change focused on the subset of people who were CU-S at their first CogState assessment and had a second CogState assessment (*n* = 257). Given the relative newness of CogState in the WRAP battery, we had few who had transitioned from CU-S to CU-D (*n* = 16) or MCI (*n* = 6) between their first and second CogState assessment. Despite the small cell sizes, we again saw a stair-step decrease in average within-person changes across those who remained CU-S at second CogState vs. those who transitioned to CU-D or MCI. For the CogState composite, the CU-D group declined approximately six points more (on a 100 point scale) than the CU-S group (Cliff’s delta effect size in upper end of small range). The MCI group decline was 14 points lower on CogState than the CU-S group (large Cliff’s delta). Effect sizes were again larger for declines in the two memory composites and the PACC composite. Interestingly, although all were CU-S at baseline with mean baseline standard scores ranging from ∼90 to 108 at that visit, those who stayed CU-S at CogState follow-up were performing better on average at first CogState for all but the Executive function composite than those who progressed to CU-D or MCI at last visit.

Prior work suggests that non-pathologic (i.e., “normal”) aging is associated with declines in executive function and processing speed, and that when these factors are taken into account, the relationship between normal aging and memory performance is greatly reduced (Salthouse, 1996). Results from this study and others support the use of learning/memory measures or multi-domain composites when examining cognitive performance in individuals early in the Alzheimer’s disease process. Together, these findings are consistent with other studies indicating that cognitive declines start well before a diagnosis of MCI or dementia (e.g., Wilson et al., 2011; Karr et al., 2018) and our results suggest that subtle declines in cognitive functioning (i.e., observable in CU-D) are detectable cross-sectionally and longitudinally prior to a diagnosis of MCI.

### Finding 3: CU-D Persons Were at Increased Risk of Progression to MCI/Dementia Compared to CU-S Persons

Although rate of progression to clinical statuses in our late-middle-aged sample was relatively low, we found consistent evidence across different definitions of “progression to clinical impairment” that CU-D was associated with increased risk of progression compared with CU-S. When examining groups at-risk for later development of dementia due to AD, the majority of research has focused on individuals with a diagnosis of MCI, as well as those with subjective memory complaints or modifiable risk factors (Mitchell and Shiri-Feshki, 2009; Mitchell et al., 2014; Cooper et al., 2015). The current findings add to the broader literature by providing additional support for the validity of CU-D as a subgroup of CU individuals who are at an increased risk of later development of clinical impairment.

Despite the strong support for an increased risk of clinical progression when examining CU-D individuals, about half of these individuals reverted to CU-S status at a subsequent visit and about half with MCI reverted to a CU group at a subsequent visit. These findings are consistent with other reports of instability in MCI within longitudinal cohort studies as compared with clinical settings. For example, in a Swedish population study of 60- to 95-year-olds, over half of those with MCI initially had reverted to unimpaired at the 6-year follow-up (Overton et al., 2019). Other studies with lower to similar reversion rates have shown that individuals who revert from MCI to CU are still at increased risk of future cognitive decline compared to those who maintain normal cognitive status (Koepsell and Monsell, 2012; Roberts et al., 2012; Aerts et al., 2017); thus, the categories of MCI and CU-D may still allow us to identify individuals at risk for subsequent decline. Future research may benefit from examining additional ways to improve the test–retest reliability of these constructs, including collecting more frequent cognitive assessments (such as those obtained from mobile devices, Kaye, 2008) or requiring subtle deficits at multiple visits (Koscik et al., 2014) or on multiple tests (e.g., Jak et al., 2009; Clark et al., 2016).

### Finding 4: PET Measures of Beta-Amyloid Plaques and Neurofibrillary Tangles Varied Across Cognitive Status Groups

Despite small n’s in our CU-D and MCI groups with PET data, we cautiously interpret our results as preliminary evidence that the CU-D group includes participants with elevated amyloid and tau PET biomarkers and longer duration of PET amyloid burden. Specifically, when using most recent PET scan and most recent cognitive status, those in the CU-D group who are positive for amyloid represent persons with AD-related “transitional cognitive decline” as described by Jack et al. (2018) in the NIA-AA research framework for AD. These results align with recently published WRAP data which showed that decline in continuous longitudinal cognition (PACC3 composite) was more rapid among those with vs. without amyloid at baseline and that longer duration of amyloid at baseline PACC3 was associated with faster decline (Koscik et al., 2020). These results also fit well with the findings from a prior meta-analysis demonstrating consistent relationships between measures of beta-amyloid deposition and both episodic learning/memory and global cognition, with less consistent findings for executive function (Hedden et al., 2013; Clark et al., 2018).

### Strengths, Limitations, and Future Directions

Strengths of this study include that our operationalization of CU-D is strongly aligned with recommendations in the literature for identifying impairment antecedent to MCI, including cognitive criteria for “preclinical AD” described by Epelbaum et al. (2017) as well as the “transitional cognitive decline” construct described recently as a stage of subtle cognitive decline in the presence of AD-related biomarkers in Jack et al. (2018). The described flagging algorithm was informed by actuarial approaches that have been shown to detect early cognitive decline (e.g., Jak et al., 2009; Koscik et al., 2014; Clark et al., 2016). The current findings indicate that an early, CU-D stage is associated with subclinical cognitive decline that is detectable in late middle-age, and that such declines are associated with increased prevalence of elevated amyloid and tau PET biomarkers and increased risk of future clinical impairment. The identification of a subclinical category and evidence of different rates of cognitive change within this category relative to CU-S and MCI have implications for clinical trial design and early intervention/secondary prevention efforts.

Limitations include the following. First, as may be expected given the relatively young age of the WRAP sample, there was a modest number of MCI, AD or other dementia cases. To fully understand how the CU-D construct aligns with dementia endpoints and the new NIA-AA A/T(N) framework (Jack et al., 2018), a larger sample of participants would be beneficial to see if amyloid and tau patterns persist, as would examining how CU-S, CU-D and MCI groups differ on additional biomarkers (e.g., neurodegeneration and vascular biomarkers). The higher proportions of men and non-white participants flagged as CU-D or MCI needs further study to understand whether clinician bias may influence status determinations. There is both ecological validity and circularity in the analyses of progression or decline since some of the tests that were used to determine baseline cognitive status (CU-S vs. CU-D) were also included when determining cognitive status at later visits; our inclusion of the CogState composite sought to minimize this circularity since it is not part of the consensus process. Finally, although clinicians reviewed longitudinal patterns in raw and standard scores from the core battery in determining cognitive status, no formal within-person change norms are currently part of the cognitive status determination. Future research will examine several additional areas including: comparing the current approach to approaches incorporating our internally derived algorithmic longitudinal change (conditional) norms (Koscik et al., 2019) to see if the CU-D can be further improved; examining whether subjective complaints of functioning are sensitive to subclinical decline in this sample; and whether factors can be identified that predict reversion to less impaired or progression to more impaired statuses.

## Conclusion

Findings from this study indicate that traditional neuropsychological data offer a means of identifying CU people who are at-risk of progressing to clinical MCI or dementia, including AD dementia. Although the current AT(N) framework emphasizes the use of biomarkers for defining the preclinical phase of the disease, the current study indicates that neuropsychological performance and informant reports can be used to define a subclinical syndrome with both concurrent and predictive validity, and that this syndrome is associated with AD biomarkers in late middle age. Although not all who meet CU-D criteria will have AD disease or will progress to clinical dementia, this group appears to be at increased risk. Future research will follow this group over time and will also examine how other variables such as CSF AD markers, genetics, and lifestyle factors differ between CU-S and CU-D. The ability to identify persons prior to reaching clinical levels of impairment has implications for clinical trial design and early intervention or prevention efforts.

## Data Availability Statement

The original contributions presented in the study are included in the article/Supplementary Material, further inquiries can be directed to the corresponding author.

## Ethics Statement

The studies involving human participants were reviewed and approved by University of Wisconsin School of Medicine and Public Health Institutional Review Board. The patients/participants provided their written informed consent to participate in this study.

## Author Contributions

RL, BH, SA, LC, EJ, KM, and SJ contributed to the conception and design of the work. RL performed data analyses and drafted the manuscript. All co-authors provided approval and contributed to interpretation of the results and critical revisions. All authors contributed to the article and approved the submitted version.

## Conflict of Interest

The authors declare that the research was conducted in the absence of any commercial or financial relationships that could be construed as a potential conflict of interest.

## Publisher’s Note

All claims expressed in this article are solely those of the authors and do not necessarily represent those of their affiliated organizations, or those of the publisher, the editors and the reviewers. Any product that may be evaluated in this article, or claim that may be made by its manufacturer, is not guaranteed or endorsed by the publisher.

## Footnotes

1 www.fil.ion.ucl.ac.uk/spm
