# Supplementary material for: Validity Evidence for the Research Category, “Cognitively Unimpaired – Declining,” as a Risk Marker for Mild Cognitive Impairment and Alzheimer’s Disease
Source: Front Aging Neurosci. 2021 Jul 26;13:688478. doi: 10.3389/fnagi.2021.688478 (PMC8350058; doi:10.3389/fnagi.2021.688478)
Supplement: Supplementary file 1 [file Data_Sheet_1.PDF]

Supplemental Table 1

Variables included in flagging algorithm	Review by consensus team if one or more of these criteria are met
**Internally derived, demographically adjusted robust norms for factor scores[1]**	
Immediate Memory and Verbal Learning and Memory Factor Scores	One or both memory factor scores more than 1.5 SD below expected at most recent visit
	Two of the last three visits having abnormal scores on one or both memory factors
Working Memory and/or Speed and Flexibility Factor Scores	more than 1.5 SD below expected at last visit
	Two of the last three visits having abnormal scores on Working Memory or Speed and Flexibility factor scores
**Informat reports[2]**	
Clinical Dementia Rating Scale Global Score (CDR)	CDR > 0 at most recent visit
Instrumental Activities of Daily Living (IADL)	IADL < 14 at most recent visit
Short Informant Questionnaire on Cognitive Decline in the Elderly (IQCODE)	IQCODE > 52 at most recent visit
**Internally derived, demographically adjusted robust norms for individual tests by cognitive domain[3]**	
	Participants can be flagged for review by evidence of within or across domain deficits as follows:
**Immediate Memory Domain**	
Rey AVLT Sum of Learning Trials	Domain specific: At least two of three test scores 1.5 SD below expected within a domain at the most recent visit;
Brief Visuospatial Memory Test-Revised (BVMT) Sum of Learning Trials	
WMRS Logical Memory I (Story A + B)	Across domains: At least one test score from three or more domains is 1.5 SD below expected at the most recent visit.
**Delayed Memory Domain**	
Rey AVLT Delayed Recall	
BVMT Delayed Recall	
Logical Memory II (Delayed story recall)	
**Executive Function Domain**	
WAIS-R Digit Symbol Substitution	
Trail Making Test Part B	
Stroop Color-Word Interference	
**Language Domain**	
Semantic fluency (Letters C, F, L)	
Phonemic fluency (Animal Naming)	
Confrontatoinal Naming (Boston Naming test)	
**Individual tests with absolute cut scores[4]**	
Clock Drawing Test (CDT) Total Score	CDT <= 7
Mini-Mental Status Exam (MMSE)	MMSE <= 26
Rey AVLT Delayed Recall	AVLT Delayed Recall <= 5
Logical Memory II (Delayed story recall)	Logical Memory II <=16

Note: Initially in 2013, the flagging algorithm included components 1 and 2 (factor score WRAP-specific norms and informant reports). In 2015, the algorithm was expanded to include components 3 and 4 (individual tests WRAP-specific norms and test-specific cut-offs for core tests). The full algorithm was used to identify records to review that had been accrued prior to the initiation of the consensus review process.
