# Supplementary material for: Validity Evidence for the Research Category, “Cognitively Unimpaired – Declining,” as a Risk Marker for Mild Cognitive Impairment and Alzheimer’s Disease
Source: Front Aging Neurosci. 2021 Jul 26;13:688478. doi: 10.3389/fnagi.2021.688478 (PMC8350058; doi:10.3389/fnagi.2021.688478)
Supplement: Supplementary file 2 [file Data_Sheet_2.PDF]

Supplemental Table 2: Diagnostic Criteria for WRAP Consensus

Cognitively Unimpaired-stable

1. If a participant is not flagged for consensus by flagging criteria, the participant will be automatically designated as cognitively unimpaired

2. If a participant is flagged and reviewed at consensus, the participant will be designated as cognitively unimpaired if:

    A. Cognitive performance is within the expected range for that individual based on all available information (may be based on clinical judgment and/or on cognitive test performance using WRAP and/or published/standard normative data). *Note: Cognitive performance may be in the impaired/abnormal range based on population norms, but performance is within the expected range for that individual. Ultimately this is a clinical decision.*

    B. May or may not report subjective cognitive complaints.

    C. No evidence of cognitive decline on longitudinal cognitive testing (if available).

Based on combination of NIA-AA 2018 framework syndromal categorical cognitive staging – Cognitively Unimpaired and numerical clinical staging – Stage 1 (Jack et al., 2018; Alzheimer's & Dementia)

Cognitively Unimpaired – Declining (formerly psychometric or early MCI)

1. Cognitive performance on the whole is broadly within the expected range on objective cognitive tests using published/standard normative data. Focal abnormal scores may be present.
2. May be some subjective cognitive complaint from participant or informant.
3. Evidence of subtle cognitive decline based on at least one of the following:

    A. Performance is below expected range (e.g., ≥ -1.5 SD below mean) based on WRAP robust normative data (AND this performance is not within the expected range for a particular individual based on all available information).

    B. Performance is within expected range using published/standard normative data, but nevertheless lower than expected for a particular individual based on all available information (e.g., an individual with a PhD with low average performance in one or more modalities/tests).

    C. There is a decline from baseline on longitudinal cognitive test performance (and the decline is not due to a reason listed in the Impaired, not MCI category).

Based on portions of NIA-AA 2018 framework numerical clinical staging – Stage 2 (Jack et al., 2018; Alzheimer's & Dementia) and discussions with WRAP clinicians. Note that in ADRC these individuals would be designated as Cognitively Unimpaired/Normal.

Mild Cognitive Impairment (formerly "clinical MCI")

1. Subjective cognitive complaint based on at least one of the following:

    A. Participant or study partner's verbal or survey reports or clinician report

    B. Quick Dementia Rating Scale (QDRS)

    C. Clinical Dementia Rating Scale (CDR)

    D. IQCODE

    E. MFQ

2. Clinician judgement of cognitive impairment in one or more cognitive domains using internal or published/standard normative data or evidence of decline in longitudinal test performance.

4. Preservation of independence in functional abilities based on at least one of the following:

    A. Participant or study partner's report to a clinician (e.g., nurse practitioner)

    B. Abnormal IADL scale score

Based on Albert et al., 2011 "The diagnosis of mild cognitive impairment due to Alzheimer's disease: recommendations from the National Institute on Aging-Alzheimer's Association workgroups on diagnostic guidelines for Alzheimer's disease" in Alzheimer's & Dementia.

**Impaired, not MCI**
1. Objective cognitive impairment using normative data but not demonstrating progressive decline from a previously higher level:<br>A. The objective impairment is likely unrelated (by history or pattern of performance) to a neurodegenerative disease and instead more likely due to another explanation: e.g., due to long-standing brain dysfunction (e.g severe brain trauma, severe anoxia, epilepsy with persisting seizures), history of a chronic disorder affecting academic achievement (ADHD/LD), long standing Axis I psychiatric condition (e.g., severely depressed, schizophrenia, bipolar disorder), medications (e.g., opioids or anticholinergic medications), delirium, substance use (e.g., daily marijuana user).<br>--This label may be used when there is unsuficient information to determine CU-D or MCI (such as at a baseline evaluation, or when missing clinician exam or other pertinent information).<br>--The label may be revised post-hoc as more information and time makes the diagnosis clearer.
Note: poor task engagement (e.g., fell asleep during testing) should not be construed as impairment; rather those test scores that are deemed invalid should be sequestered and coded as invalid.

**Dementia**
Cognitive or behavioral (neuropsychiatric) symptoms that:<br>1. Interfere with the ability to function at work or usual activities (including IADLs); and<br>2. Represent a decline from previous levels of functioning and performing; and<br>3. Are not explained by a delirium or major psychiatric disorder (in other words, not due to a non-neurodegenerative disease); and<br>4. Evidence of cognitive impairment through a combination of (A) history-taking from the patient and a knowledgeable informant and (B) objective cognitive assessment (e.g., neuropsychological testing); and<br>5. The cognitive or behavioral impairment involves a minimum of two of the following domains:<br>A. Impaired ability to acquire and remember new information—symptoms include: repetitive questions or conversations, misplacing personal belongings, forgetting events or appointments, getting lost on a familiar route.<br>B. Impaired reasoning and handling of complex tasks, poor judgment—symptoms include: poor understanding of safety risks, inability to manage finances, poor decision-making ability, inability to plan complex or sequential activities.<br>C. Impaired visuospatial abilities—symptoms include: inability to recognize faces or common objects or to find objects in direct view despite good acuity, inability to operate simple implements, or orient clothing to the body.<br>D. Impaired language functions (speaking, reading, writing)—symptoms include: difficulty thinking of common words while speaking, hesitations; speech, spelling, and writing errors.<br>E. Changes in personality, behavior, or comportment—symptoms include: uncharacteristic mood fluctuations such as agitation, impaired motivation, initiative, apathy, loss of drive, social withdrawal, decreased interest in previous activities, loss of empathy, compulsive or obsessive behaviors, socially unacceptable behaviors.
Based on McKhann et al., 2011 “The diagnosis of dementia due to Alzheimer’s disease: recommendations from the National Institute on Aging-Alzheimer’s Association workgroups on diagnostic guidelines for Alzheimer’s disease” in Alzheimer’s & Dementia.<br>Note: The differentiation of dementia from MCI rests on the determination of whether or not there is significant interference in the ability to function at work or in usual daily activities. This is inherently a clinical judgment made by a skilled clinician on the basis of the individual circumstances of the patient and the description of daily affairs of the patient obtained from the patient *and* from a knowledgeable informant.
